# Factors Affecting Disparity Between Estimated Liver Volume and Actual Liver Volume in Living Donor Liver Transplantation

**DOI:** 10.3390/diagnostics16152442

**Published:** 2026-08-02

**Authors:** Tonguc Utku Yılmaz, Emre Tunçcan, Abdurrahman Furkan Cetişli, Aylin Altan Kuş, Güzide Özdil, Ali Özer, Murat Yıldar, Hamdi Karakayalı

**Affiliations:** 1 Department of General Surgery, School of Medicine, Acibadem Mehmet Ali Aydinlar University, Istanbul 34752, Turkey; 2 School of Medicine, Acibadem Mehmet Ali Aydinlar University, Istanbul 34752, Turkey; 3 Department of Radiology, Atakent Hospital, Acibadem Mehmet Ali Aydinlar University, Istanbul 34303, Turkeyguzide.ozdil@acibadem.com (G.Ö.); 4 Organ Transplantation Unit, Atakent Hospital, Acibadem Acibadem Mehmet Ali Aydinlar University, Istanbul 34303, Turkey

**Keywords:** living donor liver transplantation, donor hepatectomy, three-dimensional volumetry, estimated graft volume, actual graft weight

## Abstract

**Background:** Volumetric evaluation is critical in living donor liver transplantation. Liver volume, depending on body weight and height, and radiological estimates have made important contributions, but some inconsistencies remain. We aimed to assess the estimated and radiological liver volumes with respect to actual graft weight and potential factors contributing to inaccuracy. **Method:** A total of 623 donors were evaluated in this retrospective study. The estimated liver volumes were calculated with the Vauthey formula. The laboratory and liver biopsy results were obtained. Three-dimensional liver tomography was used to assess liver volume. Actual graft weight was measured on the back table after perfusion. Graft types were recorded. A discrepancy of more than 10% is accepted as discordant. The possible reasons were evaluated. **Results:** The mean estimated liver volume and radiological liver volume were 1216 cm^3^ and 1290 cm^3^, respectively. The mean radiological right lobe volume and the actual right lobe graft weight were 815 and 846, respectively. Liver density, liver steatosis percentage, blood cholesterol levels, blood triglyceride levels, and blood total bilirubin levels are factors contributing to the disparity between the estimated and radiological volume differences. Vitamin B12 and albumin levels were associated with the discrepancy between radiological liver volume and actual graft weight. Underestimation was significant in the left lateral lobe. **Conclusions:** Three-dimensional volumetry is useful for size matching. Discrepancies of more than 10% are attributable to liver steatosis or splitting lines.

## 1. Introduction

Due to the shortage of cadaveric transplantation, living donor liver transplantation (LDLT) is a promising alternative for patients waiting for liver transplantation. The success of LDLT depends on the anatomy and physiology of the liver from which a partial graft would be procured. Adequate liver volume is crucial for both the donor and recipient in LDLT. Small grafts in recipients (graft-to-recipient weight ratio [GRWR] < 0.8%) are associated with graft dysfunction and increased risk of complications, including cholestasis, coagulopathy, and ascites [1]. For donors, a remnant volume of 30% is a safe threshold for safety with minimal steatosis [2]. For these reasons, estimating the donor liver volume and performing volumetric analysis of liver segments are important for both donor selection and evaluation.

The first step in evaluating donors for the recipient is to collect demographic data. To identify the most appropriate donor among donor candidates, estimating the standard liver volume is a time-saving method. Several formulas use body weight, body height, and body surface area to estimate standard liver volume [3,4,5]. These formulas can help choose the most appropriate donors, who will be searched in detail among donor candidates.

Computed tomography (CT) is the most commonly used method for preoperative liver volumetry. Although manual CT volumetry is accepted as the gold standard, it is time-consuming, and other methods, like semiautomatic and automatic methods, have also achieved acceptable accuracy [6,7]. However, some studies have reported a 5% to 15% margin of error in estimating graft volume by CT [7,8]. An error in the margin of more than 10% might lead to small-for-size syndrome or procurement of more than expected from the donor [8]. For these reasons, we sought to assess the estimated and radiological liver volumes with respect to actual graft weight and potential factors contributing to inaccuracy.

## 2. Material and Method

This retrospective cohort study was conducted at the Organ Transplantation Center of Acıbadem Atakent Hospital between November 2015 and November 2024. The study was approved by the local ethics committee (ATADEK-2025/7) in accordance with the Declaration of Helsinki. Six hundred twenty-three consecutive donors were included in the study. Donors from whom monosegment liver grafts (10 cases in total) were procured were excluded from the study. At our centers, donors aged 18 to 55 are accepted for further evaluation. In the first step, data on donor body weight (BW, Kg), body height (BH, m), and blood groups are obtained from donor candidates. Body mass index (BMI is calculated by BW/(BH ∗ BH) (kg/m^2^). Body surface area (BSA) is calculated by the square root of (BW ∗ BH)/3600$\sqrt{BW*BH/3600}$ (m^2^). If there is more than one donor candidate, we prefer to proceed with the candidate whose body mass index is less than 30 kg/m^2^ or whose estimated liver volume exceeds 1/70 of the recipient’s weight (in grams). Estimated liver volume (ELV) is calculated with the Vauthey formula (ELV = −794.41 + 1267.28 × BSA) (cm^3^) [3]. Psychiatry consultations are conducted before the onset of donor evaluation. After psychiatric approval, laboratory tests for systemic function and viral serology, and a triphasic CT evaluation for volumetry, vessel anatomy, and steatosis are performed. The minimum acceptable GRWR is 0.8, with a 30% remnant. Magnetic Resonance Cholangiopancreatography is performed to evaluate biliary anatomy. Percutaneous liver biopsy is performed in all donor candidates in our center. Macrosteatosis of more than 20% was not accepted. The same radiologist evaluated all radiological images, and the same team performed surgeries for pediatric cases with BW < 30 kg. The radiologist is requested to provide the volume of the donor’s left lateral lobe. The left lateral lobe may be large in pediatric patients with a body weight of less than 20 kg. For this reason, reduced-size left lateral lobe liver transplantation is preferred for pediatric patients less than 20 kg.

### 2.1. Donors

The donor’s preoperative evaluation is complex and performed in a stepwise manner. To save time and unnecessary evaluations, selecting a suitable donor for further evaluation is important. The donors’ age, sex, BMI, blood groups, BSA, and ELV were obtained. The patients’ preoperative laboratory blood tests including; hematocrit, platelets, glucose, creatinine, uric acid, calcium, albumin, cholesterol, triglyceride, alanine transaminase (ALT), Rheumatoid factors (RF), Antistreptolysin (ASO), aspartate aminotransferase (AST), gamma-glutamyl transferase (GGT), alkaline phosphatase (ALP), potassium, total bilirubin, ferritin, iron, Vitamin B12, folic acid, and ceruplasmin were evaluated. The graft types, radiological graft volumes, and degrees of steatosis on liver biopsy were collected. The liver biopsies were performed from the right lobe by an interventional radiologist. The liver biopsy provides the percentage of steatosis in the liver sample.

### 2.2. Computed Tomography Protocol

CT imaging was performed using a 256-slice multidetector scanner (Somatom Definition Flash; Siemens Healthcare, Forchheim, Germany). A non-contrast-enhanced abdominal scan was obtained before contrast administration. Iodinated contrast medium (1.5 mL/kg; 350 mg I/mL; Opaxol 350, Opakim, Turkey) was injected via an antecubital vein using a dual-syringe power injector (Stellant; MEDRAD) at 4–5 mL/s, followed by a 50 mL saline flush.

Precontrast, arterial, portal venous, and venous phase images were acquired with a slice thickness and increment of 3 mm at a tube voltage of 120 kV. The density of the liver and spleen was measured and expressed in Hounsfield Units (HU). If the liver parenchyma density is at least 10 HU less than the spleen’s, it is diagnostic for fatty liver.

### 2.3. CT Volumetry Protocol

A semi-automated software (Myrian^®^) was used for volumetry in all patients. After image upload, the contours of the entire liver were delineated by differentiating liver parenchyma from the surrounding adipose tissue. False-positive and false-negative segmentations were corrected using manual editing tools by an abdominal radiologist. Three-dimensional reconstruction and calculation of total liver volume were performed. The radiological transection plane was drawn along the middle hepatic vein (MHV) to delineate the right lobe (RL) and left lobe (LL) of the liver. The inferior vena cava, gallbladder, and extrahepatic and main branches of the portal vein were excluded from liver volumetry. Radiological liver volumes (RLV) were measured; total liver volume and right and left lobe volumes were calculated for right and left hepatectomy cases. For left lateral lobe volume, the radiological transection plane was drawn along the falciform ligament. Caudate lobe volume is not included in the volume analysis.

### 2.4. Surgical Technique

Donors underwent right hepatectomy, left hepatectomy, left lateral hepatectomy, or reduced left lateral hepatectomy. The decision depends on the recipient’s illness, GWRW, remnant volume, and the graft’s vascular anatomy. All donor hepatectomies were performed with laparotomy. For right hepatectomy, the right liver is mobilized, and the short hepatic veins are ligated except for the inferior hepatic vein, which is larger than 5 mm. Significant hepatic veins from segments 5 and 8 were preserved if the diameter was larger than 5 mm. The MHV is always preserved on the donor side. In left hepatectomy, the MHV is included in the graft side. For left lateral hepatectomy, the transection line is 1 cm right to the falciform ligament and continues to the anterior border of the caudate lobe. The vascular supply of the caudate lobe is ligated during left hepatectomy and left lateral lobectomy. For this reason, the caudate lobe becomes ischemic, and the caudate lobe volume is not measured during the volumetric evaluation before the operation for left hepatectomy and left lateral lobectomy. Reduced-size left lateral lobes were used for pediatric cases with a BW of less than 10 kg to prevent large-for-size syndrome. The weights of the transplanted grafts are accepted as the actual graft weight (AGW).

### 2.5. Measurement of Actual Graft Weight

After liver retrieval, the graft is flushed with HTK (histidine-tryptophan-ketoglutarate) solution (Custodiol, Dr. Franz Köhler Chemie GmbH, Absbach-Hahnlein, Germany) via the portal vein and hepatic artery until the outflow from the hepatic veins is clear at 4 °C. The liver weight is measured after trimming the liver with a portable electronic weighing machine. A total of 1 cm^3^ of liver was estimated as 1 gr.

### 2.6. Statistical Evaluation

The difference between standard liver volumes and radiological total liver volumes, and the difference between radiological graft volume and actual graft volumes, were compared. In reduced left lateral lobe grafts, the left lateral lobe graft is accepted and compared with the radiological left lateral lobe volume. If there is more than a 10% difference between the comparisons, these cases were searched for possible risk factors. The discordance rate is not estimated from previous studies, which report values ranging from 8.6% to 14.6% [9]. This estimated cutoff is not a clinical threshold but also a quality parameter for radiological liver volumetry. Patients whose AGW is 10% higher or 10% lower than RLV are evaluated.

Statistical tests were performed using NCSS (Number Cruncher Statistical System) 2020 version Statistical Software (NCSS LLC., Kaysville, UT, USA). Descriptive statistics were reported as mean, standard deviation, median, and minimum and maximum scores for quantitative variables, and frequencies and percentages for qualitative variables. The Shapiro–Wilk test, skewness and kurtosis values, and Box Plot graphs were used to assess the normality of the distribution.

A Student *t*-test was used to compare two normally distributed groups, and a one-way ANOVA was used to compare multiple groups. The Bonferroni test was used to determine the group that caused the difference.

A Bland–Altman plot was created by plotting the difference between ELV and RLV against their mean, and the 95% confidence interval (mean ± 1.96 × SD) was used to assess the level of correlation between the two methods. The same evaluation was performed for RLV and AGW.

The Mann–Whitney U test was used to evaluate variables that do not follow a normal distribution.

Pearson correlation analysis was performed to evaluate the relationships between variables.

Pearson’s Chi-Square test was used to compare qualitative data.

Results were evaluated at a 95% confidence interval, with a significance level of *p* < 0.05.

## 3. Results

**Figure 1:**
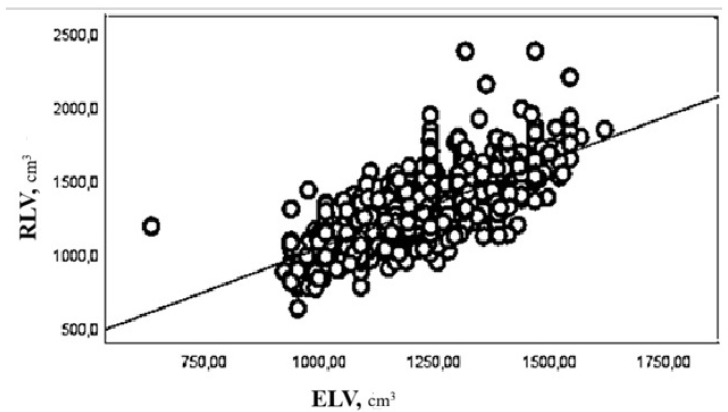
The correlation between RLV and ELV. There is a significant correlation between RLV and ELV (r = 0.684; *p* = 0.001; *p* < 0.01).

**Figure 2:**
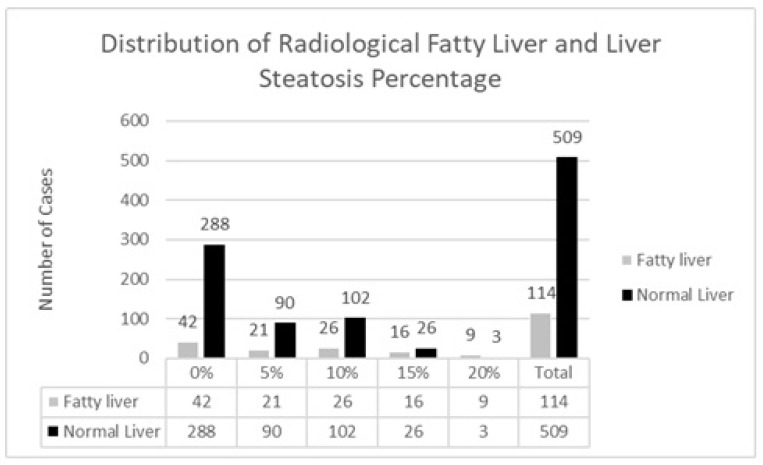
The distribution of radiological fatty liver and liver steatosis percentages. The rate of cases with more than 10% liver steatosis in radiologically fatty livers is significantly higher than the rate of cases with more than 10% liver steatosis in radiologically normal livers (*p* = 0.001). The rate of cases without steatosis in radiologically normal livers is significantly higher than in radiologically fatty livers (*p* = 0.001).

**Figure 3:**
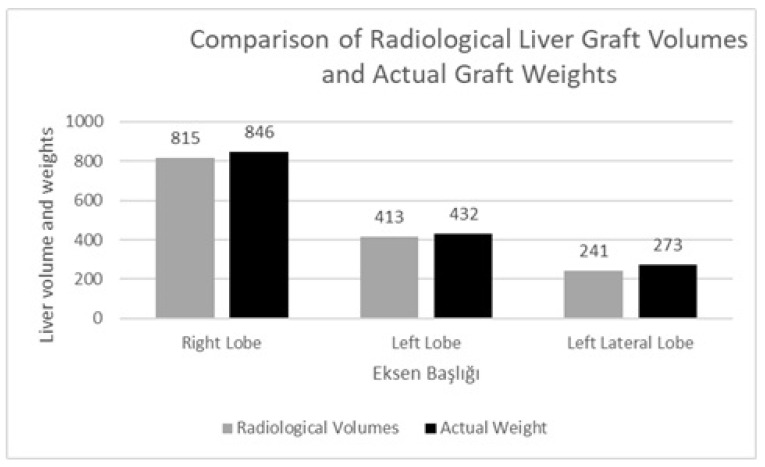
The mean cholesterol levels among liver steatosis. There is a significant correlation between the mean cholesterol levels and liver steatosis (r = 0.38, *p* < 0.01).

The same team performed a total of 623 donor hepatectomies. There is no mortality among the donors. There is no complication related to liver biopsy. The mean preoperative laboratory results were within the normal range; the mean values are provided in the Supplementary Materials. There were 286 (45.9%) females and 337 (54.1%) males. The donor characteristics and volumetry results are given in Table 1. There are significant differences between males and females according to ELV and liver steatosis percentages (Table 1). The mean radiological liver and spleen density were 59.96 ± 5.01 and 45.68 ± 3.50 HU. The blood group distribution was: 0: 334 (53.7%), A: 206 (33.1%), B: 67 (10.8%), and AB: 15 (2.4%). The correlation between RLV and ELV is given in Figure 1. There is a significant correlation between RLV and ELV (r = 0.684; *p* = 0.001; *p* < 0.01). The Bland–Altman graph showed that the 95% limits of correlation ranged from −115 to +146. A difference of more than 10% between ELV and RLV is considered preop-discordant; a difference of less than 10% between ELV and RVL is considered preop-concordant. The number of preop-concordant cases is 325 (52.1%). The number of preop-discordant cases was 298 (47.8%). The highest percentage difference is 18% (range: −13% to 18%). In the comparison of preop-concordant and preop-discordant cases, liver density, liver steatosis percentage, blood cholesterol levels, blood triglyceride levels, and blood total bilirubin levels were significantly different (Table 2). According to the logistic regression analysis, steatosis level is showing a significant model (χ = 79.451; *p* < 0.01) (explanatory power: 65.6%). The 1% increase in liver steatosis is associated with a 1.159-fold increase in preop-discordant risk (95% CI: 1118–1202). The number of cases whose RLV is higher than ELV by more than 10% is 246 (82.5%), and the number of cases whose ELV is higher than RLV by more than 10% is 52 (17.5%). Among cases with RLV higher than ELV, again, blood cholesterol levels, blood triglycerides, bilirubin, and liver steatosis are significant factors (*p* < 0.05). Among cases with ELV higher than RLV, there is no significant factor. According to the radiological evaluation, fatty liver was observed in 114 cases (18.2%). The mean HU difference in liver and spleen is 6.7 in fatty liver cases. Radiologically, the number of normal livers is 509 (81.8%). The distribution of radiological fatty liver and liver steatosis percentages is given in Figure 2. The rate of cases with more than 10% liver steatosis in radiologically fatty livers is significantly higher than the rate of cases with more than 10% liver steatosis in radiologically normal livers (*p* = 0.001). Also, the rate of cases without steatosis in radiologically normal livers is significantly higher than in radiologically fatty livers (*p* = 0.001). The mean cholesterol levels among liver steatosis are given in Figure 3. There is a significant correlation between the mean cholesterol levels and liver steatosis (r = 0.38, *p* < 0.01).

**Figure 4:**
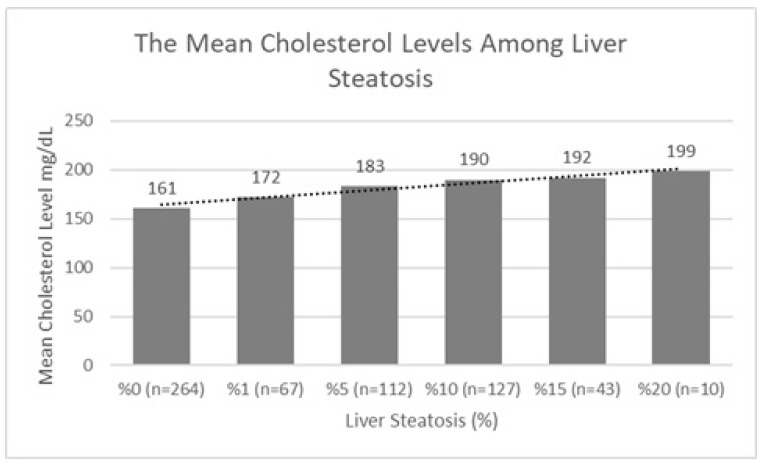
The mean radiological liver graft volumes and the mean actual graft weight. There is no significant difference between the RLV and AGW (*p* = 0.1).

**Figure 5:**
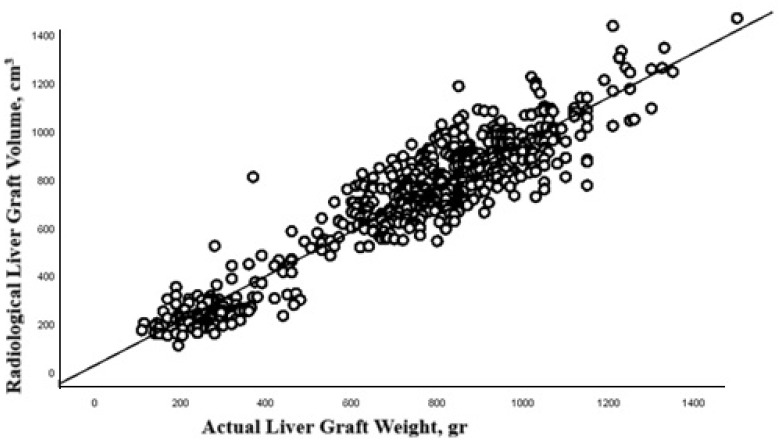
The correlation between RLV and ALW. There is a positive correlation between RLV and ALW (r = 0.932; *p* = 0.001).

The mean radiological liver graft volumes and the mean actual graft weight are shown in Figure 4. There is no significant difference between the RLV and AGW (*p* = 0.1). There is a positive correlation between RLV and ALW (r = 0.932; *p* = 0.001) (Figure 5).

A difference of more than 10% between RLV and ALW is accepted as op-discordant; a difference of less than 10% between RLV and ALW is accepted as op-concordant. Gender, BMI, fat percentage, ceruloplasmin, hematocrit, platelet count, creatinine, albumin, body temperature, and B12 levels were evaluated using backward logistic regression analysis to investigate the effect of a risk regimen where the difference between radiological graft volume and the resulting graft values exceeds 10%, grouped in different categories. Logistic Regression Analysis of Risk Factors Affecting Differences Exceeding 10% are BMI (*p* = 0.07, ODDS: 0.95, 95% CI upper and lower limits: 0.903–1.004); Ceruplasmin (*p* = 0.06, ODDS: 1.03, 95% CI upper and lower limits: 1.003–1.067); Albumin (*p* = 0.02, ODDS: 1.592, 95% CI upper and lower limits: 1.061–2.389); Vitamin B12 (*p* = 0.04, ODDS: 1.001, 95% CI upper and lower limits: 1.000–1.072). The variables included in the study were evaluated using backward logistic regression analysis. The study showed that albumin measurements, which are risk factors with an effect greater than 10%, formed a significant model (χ = 18.078; *p* = 0.001; *p* < 0.01). The explanatory power of the model is 56.0%. According to the model, a one-unit increase in albumin levels increases the risk of a difference greater than 10% by 1.592 units (95% CI: 1.061–2.389) (*p* = 0.025; *p* < 0.05). Albumin levels are independent risk factors for a difference greater than 10%. The number of concordant cases is 296 (47.5%). The number of discordant cases was 327 (52.5%). The largest percentage difference is 20%. (range −15% and +20%). The number of RLV underestimations by more than 10% is 185 cases. The distribution of underestimation was as follows: 72 (69%) left lateral lobe, five (23%) left lobe, and 108 (21.6%) right lobe. The number of RLV overestimations by more than 10% is 142. The distribution is five LL’s (23%) and 139 RL’s (28%). The underestimation rate is significantly higher in the left lateral lobe (*p* < 0.05). The Bland–Altman graph showed that the 95% limits of correlation ranged from −106 to +129. In the comparison of op-concordant and op-discordant cases, the mean levels of albumin and VitB12 differed significantly. The mean albumin levels of op-concordant and op-discordant cases were 4.26 ± 0.4 and 5.5 ± 1.3, respectively (*p* = 0.03). The mean VitB12 levels of op-concordant and op-discordant cases were 347.49 ± 134 and 371.6 ± 155, respectively (*p* = 0.01). The other laboratory tests, age, sex, and BMI did not differ significantly (*p* > 0.05). Liver HU is lower in op-discordant cases, but the difference is not statistically significant. According to the backward logistic regression analysis, albumin levels show a significant association with the outcome (χ = 18.075, *p* = 0.01) (explanatory power: 56%). Among overestimated and underestimated cases, no significant factor was associated with discordance (*p* > 0.1). For right lobes, serum albumin was associated with underestimation of RLV, and VitB12 was a significant factor associated with overestimation of RLV (*p* < 0.05). The serum albumin levels of patients with liver steatosis less than 10% and more than 10% are 4.2 g/dL and 4.1 g/dL (*p* > 0.1). The serum vitamin B12 levels of patients with liver steatosis less than 10% and more than 10% are 383 and 330 pg/mL (*p* < 0.05).

In the clinical outcomes of the underestimated cases, 16 of 72 left lateral cases underwent a reduced left lateral lobe because of a large-for-size graft. Reduction in the left lateral lobe was performed on the back table by resecting segment 3. Among clinical outcomes for overestimated cases, 40 of 139 right-lobe cases had mild or moderate small-for-size syndrome, accounting for 6.4% of all cases. The small-for-size syndrome was diagnosed with increased portal flow in intraoperative ultrasonography in 3 cases, and splenic artery ligation was performed. In the postoperative period, small-for-size syndrome was managed with splenic artery embolization in five cases, and the rest of the cases were treated with carvedilol.

## 4. Discussion

This is a retrospective, single-center study to evaluate the correlation between ELV, RLV, and AGW and to identify donor-related factors that may contribute to discordance. A total of 623 donors were included in the study, the second-highest number in a liver volume study [10]. Our study showed that ELV is an effective method for selecting the most suitable donor among candidates. The mean age of donors in our study is similar to that reported in previous studies (ranging from 27.3 to 38.35) [11,12,13]. The mean BMI of donors in our study is also similar to that reported in previous studies (ranging from 24.6 to 26.7). The mean RLV and AGW results from our study were within the ranges reported in previous studies. Our donor selection criteria are similar to those of previous studies [10,13,14,15].

Radiological liver volumetry is a reliable method for estimating the size of a suitable graft. In previous studies, a liver volume-to-weight ratio of 1.0 g/mL is the most popular ratio [11,12,13,14,15,16,17]. This is valid for nonsteatotic livers. This method is easy, reliable, and most preferred. Some authors have reported that liver density should differ from 1 g/mL as steatosis increases, but this has not gained widespread acceptance [11,18]. Each center applies its own coefficient based on its prior cases, ranging between 0.9 and 1.2 [10,11]. Our study lacks a previous correction coefficient ratio for steatotic livers. To overcome this problem, we extract the steatosis ratio from liver volume measurements at our center. The mismatch between the virtual and actual resection lines may explain the discrepancy. CT for all donor candidates may be a waste of time and unnecessary exposure to X-ray radiation. For this reason, liver volume estimation formulas can be used. In the literature, several formulas are available, including those of Heinemann, Urata, and Vautey [19,20,21]. Although this formula was derived from Japanese individuals, previous reports have also shown similar results in LDLT in Western countries. There is a linear correlation between liver volume and BSA. Although some minor errors have been observed in these formulas across different populations, they can provide useful insight into possible donor volumes [22]. Our study showed that ELV with the Vautey formula can be used to estimate liver volume. Donor preparation is a complex, multi-step evaluation process, and the main exclusion criterion is liver volumetry. For this reason, using the Vautey formula can help us select the donor candidate for further evaluation. At our center, we selected the donor for evaluation from the donor candidates using this formula and proceeded with further evaluations. In this way, we do not have to prepare all donor candidates, saving time and money. The discordance was due to elevated serum cholesterol and triglyceride levels, which are markers of fatty liver. Fatty liver likely increases the liver’s volume. Liver HU levels, biopsy steatosis, and serum cholesterol and triglycerides show that liver volume is increasing in fatty liver. Serum bilirubin levels are inversely proportional to fatty liver disease [23]. This might be the reason for the underestimation of ELV. Also, it is known that liver volume decreases after a reduction in hepatosteatosis [24]. As there is no significant difference according to sex, there is no need for a modification in the ELV formula depending on sex. BMI may not reflect fatty liver in cases with lower BMIs. The important point in living donor liver transplantation is the health of the livers of the recipient and the donor. For this reason, estimating hepatosteatosis as a percentage is useful for assessing liver health. Extracting the fatty percentage from the radiological liver volume gives us a clue about the healthy liver volume. For this reason, a liver biopsy or FibroScan is a good option for estimating the percentage of hepatosteatosis.

The radiological fatty liver definition is more than 80% accurate when compared with liver biopsies. The sensitivity and specificity of CT for liver steatosis are 72% and 88% [20]. The misdiagnosis of fatty liver on radiology might be related to differences in the contrast agent phase. Radiological estimation of liver steatosis is a reliable method. A high serum cholesterol level is an indicator of liver steatosis.

Overestimation or underestimation is the main concern in liver volumetry. We accepted 10% for discordance. This ratio is accepted based on previous studies, in which the error estimate ranges from 5% to 15% [7,8]. In the study by Kwon et al., errors exceeding 10% may lead to small-for-size syndrome [8]. In the study by Hiroshige et al., the CT error rate was 13% [25]. In previous studies, overestimation in the right lobes was mentioned. Lemke et al. advised multiplying the radiological volume by 0.75 for AGW. Niehues reported a 13% overestimation in radiologic volumetry in animal models [8,26]. In the study by Goja et al., the overestimation of RL and LL is 18.7% and 39%, respectively; the underestimation of RL and LL is 11% and 66.6%, respectively [10]. In the study by Calver et al., the overestimation was 93% in RL and 49% in LL [16]. In our study, the overestimation rate in the RL is significantly higher than in the left lateral lobe. In the literature, intraoperative blood loss or blood within the intrahepatic vasculature was thought to contribute to overestimation. Preoperative CT images show the liver’s physiological state, with fluids such as blood, bile, and lymph within it. However, the measurement of AGW is not physiological, as blood is drawn from the liver. The liver volume changed by 33% before and after the fluid infusion in the porcine model. In the study by Frericks et al., it was shown that blood volume in large vessels can be excluded from 3D volumetry, yielding more accurate results [27]. In our study, we tried to extract the main vessels as much as possible. Also, in right lobe liver transplantation, segments 5 and 8 venous orifices larger than 5 mm are reconstructed to the right hepatic vein anastomosis. However, vein orifices in segments 5 and 8 less than 5 mm might lead to congested segments, which might affect graft weight. The cause of the discordance can be corrected after implantation, allowing blood to flow to the liver again. For this reason, the blood loss hypothesis can be ruled out as not an important factor in the size discrepancy. The second reason for overestimation is the shrinkage of the liver graft after perfusion with a hyperosmolar solution [9,28]. The reduction was about 4% in a previous study [25]. To address the discrepancy between RLV and AGW arising from blood loss and liver shrinkage, several correction factors have been proposed [29]. However, the correction factors for RLV may introduce errors due to inter-individual differences.

Albumin levels are a good indicator of the severity of liver disease [30]. However, in healthy patients, there is no data on the relationship between serum albumin levels and liver density. Serum albumin levels depend not only on liver synthesis but also on distribution and on other serum components, such as cholesterol. Albumin plays an important role in cholesterol transfer between cells [31]. For this reason, albumin increases with increases in serum cholesterol in healthy people. However, in nephrotic or cirrhotic patients, the serum cholesterol and albumin levels are inversely proportional [31,32]. In our study, serum albumin levels did not reflect any abnormal clinical findings in our patients. That is why these values are only numeric results and not enough to reach a clinical outcome. In the liver, VitB12 is released from hepatocytes into the serum, thereby elevating serum VitB12 levels [33]. In the study by Koplay et al., VitB12 levels were shown to be decreased in liver steatosis [34]. However, in a recent Meta-analysis, there was no association between liver steatosis and serum vitamin B12 levels [35]. Vitamin B12 is needed as a coenzyme for the conversion of methylmalonyl-CoA to succinyl-CoA. If there is low Vitamin B12, accumulation of methylmalonyl-CoA will lead to lipogenesis and increase cholesterol levels [36]. The possible mechanism of Vitamin B12 is related to the cholesterol mechanism. However, none of our patients experienced Vitamin B12 deficiency.

**Figure 6:**
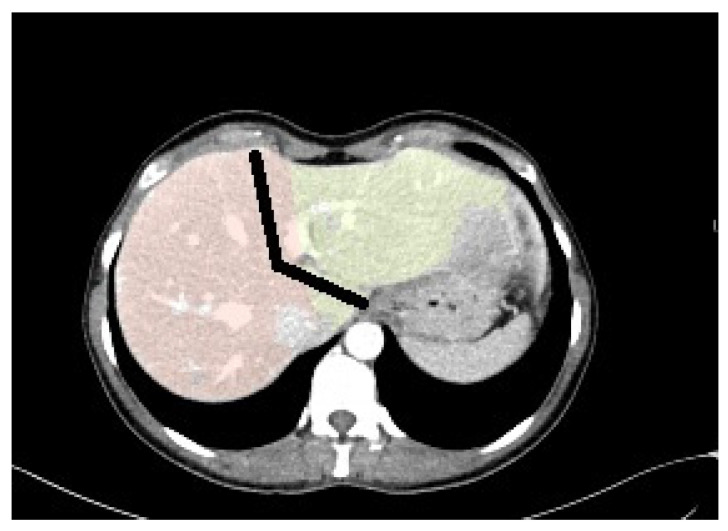
The volume of the left lateral lobe measured by the radiologist is shown in color. The actual split line is shown with a black line.

Radiological slicing can be performed under the guidance of donor surgeons (Figure 6). Thin-slice CT images, especially those with slice thicknesses of less than 2.5 mm, might be beneficial for accurate transection line and CT volumetry [13]. The high underestimation of the left lateral lobe mainly depends on the transection lines being drawn slightly right of the falciform ligament. This might be overcome by drawing the transection line 2 cm to the right of the falciform ligament and radiological measurement of the left lateral lobe (Figure 6). This finding is similar to previous studies [13]. The caudate lobe might contribute to volume discrepancies, particularly in the left caudate and left lateral caudate lobes [13]. However, the extraction of the caudate lobe volume can be easily performed in radiological volumetry.

The study has some limitations, including its single-center retrospective design. Also, liver density is typically set at 1 g/mL, but this value can be adjusted based on the degree of liver steatosis. There are new non-invasive methods for assessing liver steatosis, such as FibroScan and Magnetic Resonance Elastography, that provide alternatives to liver biopsy. These methods can be used for future studies.

As a result, three-dimensional volumetry is useful for size matching. Discrepancies of more than 10% are attributable to liver steatosis or splitting lines. Radiological slicing can be performed under the guidance of donor surgeons. Segmental changes in liver density and radiological liver volume correction factors based on laboratory results are subjects for future studies.

## Figures and Tables

**Table 1:** Demographic findings of donors and volumetry results of grafts.

	Values
Sex (n) (Male/Female)	337/286
Age (years)	32.95 ± 9.21
BMI (kg/m^2^)	24.47 ± 3.19
BSA (m^2^)	1.77 ± 0.18
Liver graft lobe (n)	
*-Right lobe*	498 (79.9%)
*-Left lobe*	21 (3.4%)
*-Left lateral lobe*	88 (14.1%)
*-Reduced size*	16 (2.6%)
Estimated liver volume in cm^3^, (median ± SD)	1216.30 ± 139.81
Male *	1255.03 ± 137.52
Female *	1170.65 ± 128.44
Radiological liver volume in cm^3^, (median ± SD)	1290.81 ± 241.66
Male	1275 ± 157.52
Female	1195 ± 132.52
Radiologic graft volume in cm^3^, (median ± SD)	
*-Right lobe*	815.36 ± 165.98
*-Left lobe*	413.86 ± 145.93
*-Left lateral lobe*	241.34 ± 65.82
**Liver Steatosis (%)**	
Male **	5.17 ± 5.61
Female **	3.53 ± 4.81
**Actual graft weight in gr, (median** ± SD**)**	
***-Right lobe***	846.26 ± 162.29
***-Left lobe***	432.14 ± 88.31
***-Left lateral lobe***	273.07 ± 53.04
***-Reduced size***	164.06 ± 30.40

SD: Standard deviation; HU: Hounsfield Unit; BMI: body mass index; BSA: body surface area. Statistical analysis: *: Student *t*-test, *p* = 0.001; **: Mann–Whitney U test, *p* = 0.001.

**Table 2:** The factors affecting the disparity between the estimated liver volume and the radiological liver volume.

Variable	Concordant (N = 325)	Discordant (N = 298)	*p*
Steatosis (%), Mean ± SD Median (Min-Max)	2.65 ± 3.65 1 (0–15)	6.34 ± 6.13 5(0–20)	**^b^ 0.001**
Triglycerides, Mean ± SD Median (Min-Max)	92.1 ± 50.1 80 (21–348)	112.5 ± 69.5 105 (25–400)	**^b^ 0.001**
Cholesterol, Mean ± SD Median (Min-Max)	170.5 ± 37.2 167 (21–282)	179.8 ± 39.8 176 (21–336)	^a^ **0.003**
Total bilirubin, Mean ± SD Median (Min-Max)	0.68 ± 0.40 0.6 (0.2–3.4)	0.85 ± 4.33 0.72 (0.1–5)	**^b^ 0.002**
Liver HU, Mean ± SD Median (Min-Max)	60.45 ± 4.83 61 (45–72)	59.43 ± 5.16 60 (38–73)	^a^ **0.011**
Age (y), Mean ± SD Median (Min-Max)	32.6 ± 9.1 32 (18–55)	33.3 ± 9.3 32.5 (18–55)	^a^ 0.710
BMI (kg/m^2^), Mean ± SD Median (Min-Max)	24.3 ± 3.0	24.6 ± 3.4	^a^ 0.241
Sex (% F)	45.2%	46.6%	^c^ 0.724

HU: Hounsfield Unit; Statistical tests: ^a^ Student *t*-test, ^b^ Mann–Whitney U test, ^c^ Pearson’s Chi-Square test.

## Data Availability

The data that support the findings of this study are available from the corresponding author upon reasonable request.

## Supplementary Materials

The following supporting information can be downloaded at: https://www.mdpi.com/article/10.3390/diagnostics16152442/s1, Table S1: The mean laboratory values of donors.

## Abbreviations

AGW	Actual graft weight
ALP	Alkaline Phosphotase
ALT	Alanine transaminase
ASO	Antistreptolysin
AST	Aspartate aminotransferase
BH	Body height
BMI	Body mass index
BSA	Body surface area
BW	Body weight
CT	Computer tomography
ELV	Estimated liver volume
GGT	Gamma-glutamyl transferase
GRWR	Graft-to-recipient weight ratio
HU	Hounsfield Units
HTK	Histidine-tryptophan-ketoglutarate
MHV	Middle hepatic vein
LDLT	Living donor liver transplantation
LL	Left lobe
RF	Rheumatoid factor
RL	Right lobe
RLV	Radiological liver volumes
VitB12	Vitamin B12
