# Supplementary material for: Factors Affecting Disparity Between Estimated Liver Volume and Actual Liver Volume in Living Donor Liver Transplantation
Source: Diagnostics (Basel). 2026 Aug 2;16(15):2442. doi: 10.3390/diagnostics16152442 (PMC13464871; doi:10.3390/diagnostics16152442)
Supplement: Supplementary file 1 [file diagnostics-16-02442-s001.zip › diagnostics-4420803-supplementary-ap.pdf]

Tabe S1. The mean laboratory values of donors.

| | Mean±SD | Median (Range) |
|---|---|---|
| **Hematocrit** | 42,14±4,71 | 42,5 (14,9-54,8) |
| **Platelet** | 259,68±61,69 | 252 (54-520) |
| **Glucose** | 92,16±13,11 | 91 (13-232) |
| **Creatinine** | 0,92±2,56 | 0,8 (0,4-59) |
| **Uric Acid** | 4,64±4,78 | 4,4 (1,3-119) |
| **Calcium** | 11,20±39,81 | 9,3 (0,5-983) |
| **Albumin** | 4,91±9,72 | 4,3 (2,9-188) |
| **Cholesterol** | 174,91±38,72 | 172 (19-336) |
| **Triglyceride** | 101,8±60,94 | 86 (0,3-400) |
| **ALT** | 27,03±13,56 | 24 (7-131) |
| **AST** | 19,14±6,24 | 18 (4,7-62) |
| **GGT** | 25,92±19,19 | 22 (2-324) |
| **ALP** | 75,16±27,46 | 72 (0,7-429) |
| **Potasium** | 4,2±1,26 | 4,1 (0,4-27,7) |
| **Total bilirubin** | 0,76±3,01 | 0,5 (0,1-75) |
| **Iron** | 84,96±44,39 | 78,5 (4,6-370) |
| **Ferritin** | 56,42±54,97 | 38,5 (1-366) |
| **Vitamine B12** | 360,11±146,53 | 339 (4,4-2000) |
| **Folic asid** | 9,61±15,56 | 8 (1,1-380) |
| **ASO** | 151,88±139,19 | 108 (0,2-1129) |
| **Ceruloplasmin** | 24,76±5,70 | 23,9 (0,8-54,6) |
| **RF** | 9,68±16,70 | 8,4 (8-392) |

SD: standart deviation, ALT: (alanine transaminase), RF: (Rheumatoid factors), ASO: (Antistreptolysin), AST: (aspartate aminotransferase), GGT: (gamma-glutamyl transferase), ALP: (alkaline phosphatase)
